# Task Dominance Determines Backward Inhibition in Task Switching

**DOI:** 10.3389/fpsyg.2017.00755

**Published:** 2017-05-10

**Authors:** Kerstin Jost, Vera Hennecke, Iring Koch

**Affiliations:** ^1^Institute of Psychology, RWTH Aachen UniversityAachen, Germany; ^2^Department of Psychology, Medizinische Hochschule BrandenburgNeuruppin, Germany; ^3^Institute of Educational Psychology, Leibniz University of HanoverHanover, Germany

**Keywords:** task switching, backward inhibition, n-2 task repetition costs, stimulus-response compatibility, task dominance

## Abstract

Switching between tasks is assumed to be accompanied by inhibiting currently irrelevant, but competing tasks. A dominant task that strongly interferes with performing a weaker task may receive especially strong inhibition. We tested this prediction by letting participants switch among three tasks that differ in dominance: a location discrimination task with strong stimulus–response bindings (responding with left-hand and right-hand button presses to stimuli presented left or right to the fixation cross) was combined with a color/pattern and a shape discrimination task, for which stimulus–response mappings were arbitrary (e.g., left-hand button press mapped to a red stimulus). Across three experiments, the dominance of the location task was documented by faster and more accurate responses than in the other tasks. This even held for incompatible stimulus–response mappings (i.e., right-hand response to a left-presented stimulus and vice versa), indicating that set-level compatibility (i.e., “dimension overlap”) was sufficient for making this location task dominant. As a behavioral marker for backward inhibition, we utilized n-2 repetition costs that are defined by higher reaction times for a switch back to a just abandoned and thus just inhibited task (ABA sequence) than for a switch to a less recently inhibited task (CBA, n-2 non-repetition). Reliable n-2 task repetition costs were obtained for all three tasks. Importantly, these costs were largest for the location task, suggesting that inhibition indeed was stronger for the dominant task. This finding adds to other evidence that the amount of inhibition is adjusted in a context-sensitive way.

## Introduction

Many everyday-life situations require the coordination of different tasks and goals. In this regard, the task-switching paradigm has become a popular tool to study those processes that enable the flexible adjustment to changing task requirements. In a typical task-switching experiment, participants switch between two (or more) tasks, which usually goes along with costs, that is, response times (RTs) and often also error rates are higher when a switch from one task to the other is required than when the task stays the same across consecutive trials. These switch costs indicate that switching even between simple tasks is not trivial and seems to require time-consuming control processes that enable cognitive flexibility (for reviews, see Kiesel et al., 2010; Vandierendonck et al., 2010).

What exactly are the mechanisms that enable flexible switching from one task to another? It is now widely accepted that part of the switch costs reflect processing demands involved in changing/updating task-specific configurations or task sets (e.g., Rogers and Monsell, 1995; Meiran, 1996), but also that proactive interference from previous settings (or task-set inertia as termed by Allport et al., 1994), contributes to the switch costs as well (e.g., Goschke, 2000; for review see Kiesel et al., 2010; Vandierendonck et al., 2010). Therefore, besides reconfiguring the system to new task requirements, “getting rid” of previous configurational settings likely also plays a role. One mechanism thought to facilitate flexible switching is inhibition: strong competitor tasks or tasks that were relevant previously constitute a source of interference and task-set carry-over. Inhibiting these competitor tasks reduces conflict and enables one to efficiently perform the currently relevant task (Mayr and Keele, 2000; see Koch et al., 2010, for a review).

The role of inhibiting or suppressing no longer relevant task sets is addressed in many theories and accounts on task switching (see Koch et al., 2010, for a review). For instance, Allport et al. (1994) suggested that when performing a task, the tendency to perform a no longer relevant and competing task needs to be suppressed or inhibited (see also e.g., Goschke, 2000). Moreover, Mayr and Keele (2000) proposed a hypothetical mechanism termed “backward inhibition” that functions as “… a counterforce to the persistent-activation property of control settings and would thus “clear the slate” for currently relevant task sets” (Mayr and Keele, 2000, p. 5). The research question we address in the present paper is whether the amount of inhibition is adjusted to the degree of automatization of a task and the influence (conflict) a task exerts on other tasks.

First evidence that inhibition plays a role in task switching comes from a finding known as switch-cost asymmetry. At the same time, asymmetric switch costs also indicate that some tasks need to be inhibited more strongly than other ones. Allport et al. (1994) observed that switch costs are higher when participants switch to the stronger, more dominant task of a pair of tasks. For instance, when participants switch between reading the word and naming the print color of incongruent color–word Stroop stimuli (e.g., the word “red” printed in green color), switch costs are higher for word reading than for color naming. Within the task-set inertia account, this, at first glance, counterintuitive effect has been interpreted in terms of inhibition: To enable performing the weaker, color-naming task the competing, normally dominant word-reading task must be actively suppressed. When a switch is now required from color naming to word reading, residual inhibition should still be present, which hampers the reactivation and/or processing of the word-reading task. Similar asymmetric switch costs have been observed for language switching in bilingual naming tasks (i.e., larger costs for switching back to the dominant of two languages, see, e.g., Meuter and Allport, 1999; Philipp et al., 2007; see Declerck and Philipp, 2015, for a review). However, the theoretical conclusiveness of such asymmetrical switch costs with respect to an underlying inhibitory mechanism remains debatable (see Koch et al., 2010; Gade et al., 2014; Declerck et al., 2015, for discussion).

Today the least controversial and widely accepted way to test inhibition in sequential task control is the assessment of n-2 task repetition costs (Mayr and Keele, 2000; see Koch et al., 2010, for a review). In this variant of the task-switching paradigm, participants switch among three tasks. The basic idea is that when switching to a new task is mediated by inhibiting no-longer relevant tasks, switching back to a just abandoned task should result in decreased performance, because inhibition persists over time and this residual inhibition needs to be overcome. The typical finding is that RTs are slower when returning to a recently abandoned task (e.g., as in AB*A* compared to CB*A* sequences). These n-2 repetition costs have been replicated many times and are to date robust against alternative interpretations (see Koch et al., 2010, for review). They, therefore, represent a widely accepted empirical marker for inhibition.

There is already some evidence that n-2 repetition costs are sensitive to the degree of task competition. For example, Schuch and Koch (2003) used a go/no-go variation and found that previous tasks are only inhibited if the current task requires a response (i.e., go trial) but not if it turned out to be a no-go trial. Moreover, Gade and Koch (2007) manipulated the representational overlap of the response sets across the tasks and found that n-2 repetition costs were largest if there was full overlap of response sets across all tasks. In another study, Gade and Koch (2005) manipulated the intertrial interval (specifically, they varied the response–cue interval in cued task switching) and found that n-2 repetition costs were largest if the preceding interval was very short, suggesting that strong residual activation of the preceding task triggers stronger backward inhibition. Finally, in language-switching studies larger n-2 repetition costs were observed for the dominant, first language (e.g., Philipp et al., 2007; Declerck et al., 2015).

Since in research on task switching it is common practice to aggregate across tasks, evidence regarding the relation between the dominance pattern of the tasks included in a switching situation and the mechanisms applied to control the impact of each task is rather scarce. Specifically, apart from language-switching studies, in which performance is typically examined for each language separately, there is hardly any evidence for the modulation of n-2 repetition costs by task dominance. One notable exception is the study of Arbuthnott (2008). She examined switching among three different digit-categorization tasks that vary in difficulty. Participants judged whether a given digit was larger or smaller than 5 (easy), odd or even (easy/intermediate), or a prime number or not (hard). In two experiments, involving either separate or overlapping response sets, larger n-2 repetition costs were observed for the easier of two tasks than for the harder one. However, this pattern was significant only in the first experiment and only if the tasks with the greatest difference in difficulty were compared, so that it cannot be considered as confirmed that the amount of inhibition targeted against an unwanted task specifically depends on the amount of competition this particular task exerts. In the present study, we, therefore, systematically manipulated task dominance and assessed n-2 repetition costs as behavioral marker for inhibitory processes separately for each task across a series of three experiments.

Task dominance was manipulated by introducing a task with high spatial stimulus–response (S–R) compatibility (Kornblum et al., 1990). In this *location task*, the participants responded with left or right button presses to objects presented left or right to the fixation cross. According to the taxonomy of Kornblum et al. (1990), this task is characterized by overlap between the relevant stimulus dimension and the response dimension – the spatial position – and, accordingly, should be performed particularly easily, so that we consider it dominant in the context of the two other tasks. Specifically, this location task was combined with two other tasks, for which stimulus and response dimensions did not overlap (i.e., to indicate the color or the shape of a stimulus with a lateralized response). Because there was no dimensional S–R overlap for these tasks, any mapping of a stimulus to a response was arbitrary, so that there should be no automatic response activation.

With this set of tasks, we assumed that with multidimensional stimuli (i.e., varying in location, color, and shape), efficient performance in a less dominant task may only be possible by inhibiting the dominant location task, because otherwise the spatial location of the stimulus would automatically activate its corresponding response. As a result, switching to the dominant location task should be impaired because of residual inhibition. A similar degree of inhibition might not be necessary for the two other tasks. If the more dominant location task indeed needs to be inhibited more strongly to efficiently perform the less dominant color and shape tasks, then n-2 repetition costs should be larger for the location task than for the other tasks.

## Experiment 1

### Materials and Methods

#### Participants

Twenty students of the RWTH Aachen University (16 female, 4 male) participated. All participants reported having normal or corrected-to-normal vision, and were naïve with respect to the purpose of the study. Mean age was 21.5 years (age range 18–28 years).

This study (experiments 1–3) was carried out in accordance with the ethical guidelines of the German Psychological Society (Deutsche Gesellschaft für Psychologie) with written informed consent from all subjects. Ethical review and approval was not required for this study in accordance with the national and institutional guidelines. All subjects gave written informed consent in accordance with the Declaration of Helsinki.

#### Stimuli, Task, and Procedure

The stimulus material consisted of visually presented multivalent objects that varied on three different dimensions with two values each: location (presented to the left or right side of the screen), color (red or blue), and shape (circle or square). Stimuli were presented on a black screen. The circles had a diameter of 2.25 cm and the squares a side length of 2 cm. Viewing distance was approximately 60 cm.

**FIGURE 1:**
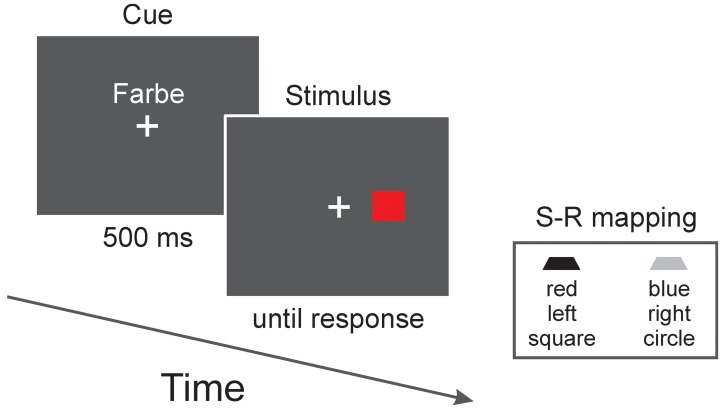
**Stimulus sequence of a trial.** The cue presented in advance indicated the relevant stimulus dimension. In this example, color is relevant (as indicated by the German word “Farbe” which means color) and, according to the stimulus–response (S–R) mapping (shown is one out of eight possible mappings), the correct response is a left-button press. Note that for the location task, only compatible S–R mappings were used in Experiments 1 and 2 and only incompatible mappings were used in Experiment 3. Furthermore, for Experiments 2 and 3 the color task was replaced by a pattern task (i.e., objects were filled with either horizontal or vertical black/white lines).

Participants had to switch among the location, color, and shape tasks. A cue presented in advance indicated the relevant dimension (see **Figure 1**). These cues consisted of the German words “Ort” (location) for the location task, “Farbe” (color) for the color task, and “Typ” (type) for the shape task^1^. The cues were presented in white color slightly above the fixation cross. Font was Arial and height was 2 cm.

Each trial started with the presentation of the task cue for 500 ms. The stimulus directly followed the cue (cue–stimulus interval thus was 500 ms). The stimulus remained on the screen until the participant responded (speed as well as accuracy were stressed in the instruction). Feedback was immediately provided after an error (the German word “falsch,” which means wrong, was presented for 500 ms). The intertrial interval was 500 ms.

Participants responded by pressing one of two response keys (the two “Alt” keys located to the left and right of the space bar). With two values for each of the three tasks, eight different mappings were possible (e.g., circle, red, and left mapped to the left hand and square, blue, and right mapped to the right hand). In Experiment 1, we only implemented those four mappings that contained the compatible S–R mapping for the location task, that is, for the location task, all subjects responded with a left hand press when the stimulus was presented left and with a right hand press when the stimulus was presented on the right. The mappings were fully counterbalanced across participants.

The experimental session started with a practice block containing 32 trials. The main part consisted of eight blocks with 96 trials each (plus two block-starting trials). Tasks switched in pseudorandom order such that each trial was a switch trial and ABA and CBA sequences occurred equally often for each task within a block. Stimuli were also assigned pseudorandomly, that is, within a block each stimulus occurred equally often in the context of each task–sequence combination and direct stimulus repetitions from one trial to the next were omitted (n-2 stimulus repetitions, however, were allowed). Blocks were separated by short breaks in which feedback about the total number of errors in the completed block was provided.

#### Design

Independent variables were the task in the current trial n, which was location, color, or type, and the task sequence which was either of the sort of n-2 repetitions (ABA) or n-2 switches (CBA). Accordingly, we ran analyses of variance (ANOVAs) with these two independent variables on RTs and error rates. *F* statistics were Greenhouse-Geisser-corrected. The uncorrected degrees of freedom, the corrected *p*-value, and the respective GG-*ε* values are reported. In the first step, we report the findings of the main effect of task in order to address the issue of task dominance. In the second step, we address inhibition and potential differences in the amount of inhibition across the three different tasks by means of the main effect of sequence and the interaction Task × Sequence. Besides the raw n-2 repetition costs (the RT difference between ABA and CBA sequences), we also calculated proportional scores (by taking performance in the CBA sequences as baseline) to account for differences in absolute RTs across the tasks.

### Results and Discussion

The first two trials of each block were removed. Trials with RTs shorter than 200 ms or above three standard deviations of a participant’s mean in each task were defined as outliers and excluded from the analyses. For RTs, only correct trials were analyzed. Moreover, only trials, in which the correct response was given in the two previous trials, were included in the analyses of RTs and error rates.

#### Task Dominance

**FIGURE 2:**
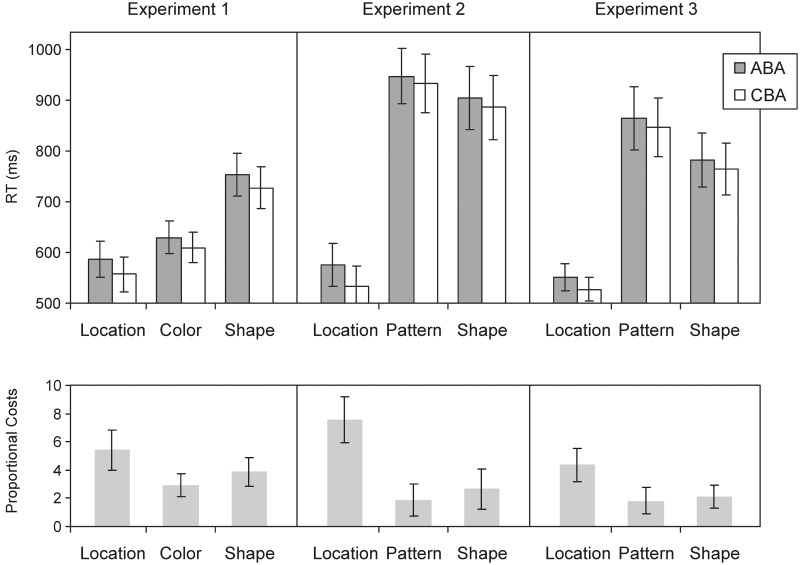
**(Top)** Response times (RTs) in Experiments 1–3 as a function of task and sequence. **(Bottom)** Proportional scores of the n–2 repetition costs (proportional RT differences between ABA and CBA sequences) calculated to account for basic RT differences across the tasks. Error bars indicate one standard error of the mean. In all three experiments, RTs are smaller in the location than in the other tasks reflecting the “dominance” of the location task. In addition, robust n–2 repetition costs are also evident in all three experiments, that is, RTs are longer when the task from trial n–2 was repeated (ABA sequence) than when the task switched. Importantly, this effect was larger for the location than for the other tasks. This especially holds when the n–2 repetition costs are set in relation to the absolute RTs in each task (see the proportional scores in the lower row of the figure).

The upper panel in **Figure 2** shows mean RTs for correct responses in the different tasks and transition sequences. As can be seen, RTs differed substantially across the tasks. This was confirmed by the main effect of task, *F*(2,38) = 66.35, *MSE* = 4,540, *p* < 0.0001, GG-*ε* = 0.911. With an average RT of 572 ms, the location task was processed significantly faster than the color and shape tasks with 620 and 740 ms, respectively, *p*s ≤ 0.001. A similar pattern was observed for the error rates: the ANOVA yielded a main effect of task, *F*(2,38) = 5.22, *MSE* = 8.98, *p* = 0.0155, GG-*ε* = 0.819. Error rates in the location task amounted to 2.10% and were smaller than in the color task with 3.71% and the shape task with 4.16%, with *p*s = 0.0590 and 0.0049. This overall pattern indicates that our experimental manipulation worked and provides evidence for the dominance of the location task over the other two tasks.

#### Backward Inhibition

Response times were smaller in ABA than in CBA sequences, replicating the typical n-2 repetition costs. The main effect of sequence was significant, *F*(1,19) = 40.56, *MSE* = 463, *p* < 0.0001. Regarding the main question in our study, that is, whether these costs are larger for the most dominant task, n-2 repetition costs indeed turned out numerically larger in the location than in the other two tasks. When we scale the difference scores (to take the huge RT differences into account when interpreting the size of the n-2 repetition costs), the 29-ms effect in the location task is equivalent to a 5.24% RT increase in the ABA compared to CBA sequences. In the other two tasks, the effects correspond with 20 and 26 ms to increases of 2.95 and 3.89%, respectively (see lower panel of **Figure 2**). Although this pattern overall meets our expectations, the interaction Task × Sequence was not significant, *F* < 1. This also holds when the proportional scores were compared across the three different tasks, *F*(2,38) = 1.13, *MSE* = 27, *p* = 0.3340, GG-*ε* = 0.780.

Sequence had no reliable effect on the error rates. Neither the main effect nor the interaction was significant (*F*s < 1).

One reason for the small and not significant differences in the size of the n-2 repetition costs might be that the effect of the dominance manipulation was not strong enough. As can be seen from **Figure 2**, RTs were also relatively small for the color task, which also significantly differed from the most difficult task, the shape task (*p* < 0.05). Moreover, 6 out of the 20 participants even did not show shorter RTs for the location than for the color task. Thus, the advantage of the location over the color task is not very clear. In Experiment 2, we, therefore, replaced the color task by a more difficult task to see if we can replicate the ordinal pattern of task inhibition effects in a more pronounced manner than in Experiment 1.

## Experiment 2

### Materials and Methods

#### Participants

Data were collected from 20 new participants of the same student population as in Experiment 1. One participant was excluded because of very slow responses (RT > 3000 ms in most of the trials) and, therefore, another new participant was run in exchange. The final sample comprised data of 9 women and 11 men. Mean age was 23.8 years (age range 19–30 years).

#### Stimuli, Task, and Procedure

Stimuli and task were the same as in Experiment 1, except for the fact that the color task was replaced by a pattern task that we assumed to be more difficult. More precisely, the two objects were now filled with either horizontal or vertical lines (black/white). Both color and pattern tasks require attending the filling of an object (and are similar in this regard). The color task, however, might be easier because of the high distinctiveness of the used colors. The tasks were cued by the German words “Ort” (location) for the location task, “Muster” (pattern) for the pattern task, and “Form” (shape) for the shape task (note that the shape-task cue also differed from the one used in Experiment 1).

### Results and Discussion

#### Task Dominance

Data trimming and analyses were performed as before. As suggested by **Figure 2**, the pattern task here was much more difficult than the color task in Experiment 1 (*p* < 0.0001 for the task comparison of the two experiments). Thus, exchanging the tasks was effective. Again, performing the location task was the fastest. The ANOVA on RTs revealed a significant main effect of task, *F*(2,38) = 78.80, *MSE* = 22,594, *p* < 0.0001, GG-*ε* = 0.716. RTs in the location task were with on average 555 ms significantly shorter than in the pattern task (941 ms) and in the shape task (895 ms), *p*s < 0.01. Moreover, this location dominance was present in each single participant with an advantage in mean RTs of at least 90 ms.

Participants also committed fewer errors in the location task (1.24%) than in the pattern and shape tasks (5.43 and 4.07%). Both the main effect task, *F*(2,38) = 17.83, *MSE* = 10.25, *p* < 0.0001, GG-*ε* = 0.708, and the direct comparisons (*p*s < 0.0001) were significant.

All in all, combining the location and shape tasks with the new pattern task yielded a much more pronounced dominance pattern than in Experiment 1. Exchanging the color task by the pattern task, apparently also had an effect on the shape task. The direct comparisons of the two experiments yielded significantly larger shape-task RTs in Experiment 2 than in Experiment 1, *F*(1,38) = 4.23, *MSE* = 112,809, *p* = 0.0466. The different cues used for indicating the shape task (the German words “Typ” versus “Form”) might be the reason for the RT differences. However, it is more plausible that feature (i.e., edge) detection for discriminating the different shapes is much easier when the objects are colored than when they are shaded. Regardless of the specifics, however, the findings from Experiment 2 suggest that the location task is clearly the dominant one when performed in the context of pattern and shape tasks.

#### Backward Inhibition

As in the first experiment, ABA and CBA sequences differed significantly in RTs, *F*(1,19) = 12.70, *MSE* = 1,399, *p* = 0.0021, for the main effect of sequence. ABA sequences were processed more slowly in all tasks and the respective difference was with 40 ms larger in the location task than in the other two tasks (14 and 18 ms for the pattern and shape task, respectively). Although the interaction Task × Sequence did not reach significance when using the raw RTs, *F*(2,38) = 1.72, *MSE* = 1,303, *p* = 0.2028, GG-*ε* = 0.683, the ANOVA on the proportional scores (taking the RT differences across tasks into account), yielded significant differences, *F*(2,38) = 5.33, *MSE* = 36, *p* = 0.013, GG-*ε* = 0.852. N-2 repetition costs in the location task differed significantly from the costs in the pattern task (*p* < 0.001) and in the shape task (*p* = 0.038).

In error rates, neither the main effect of sequence, *F*(1,19) = 3.49, *MSE* = 1.39, *p* = 0.0772, nor the interaction Task × Sequence *F*(2,38) = 2.96, *MSE* = 3.10, *p* = 0.0850, GG-*ε* = 0.697, were significant. Descriptively, n-2 repetition costs were observed only for the pattern task (with 6.16% for ABA and 4.69% for CBA sequences).

To sum up, n-2 repetition costs in RTs were larger for the dominant location task. This pattern is in accordance with our hypothesis and suggests that the location task receives more inhibition, presumably to avoid interference on the weaker tasks. As argued in the Introduction, S–R compatibility was assumed to be a relevant factor behind task dominance. The location task, as it was implemented in the two experiments so far, was characterized by compatibility both on the element level as well as on the set level: the relevant dimensions of stimuli and responses (spatial location) did overlap and, in addition, the specific mapping between a stimulus and a response was compatible (i.e., left stimulus to a left response), which might have activated spatially corresponding responses in a more or less automatic fashion. To examine whether our task dominance effect on n-2 repetition costs is driven by the more general dimensional overlap with spatial stimulus and response set or by the existence of automatic response activation with spatially corresponding S–R mappings, we used spatially incompatible S–R mappings for the location task in Experiment 3.

## Experiment 3

### Materials and Methods

#### Participants

Twenty new students (15 female, 5 male) participated. None of them took part in the previous experiments. Mean age was 23.2 years (age range 18–31 years).

#### Stimuli, Task, and Procedure

Stimuli and tasks were the same as in Experiment 2. The only change was that in this experiment the S–R mapping for the location task was incompatible, that is, participants responded in the location task with a button press on the right when the stimulus was presented left and vice versa. The four possible mappings were counterbalanced across participants. Everything else was as before.

### Results and Discussion

#### Task Dominance

As obvious from the figure, the location task was with a mean RT of 540 ms again processed much faster than the other two tasks with 856 and 773 ms. The main effect of task, *F*(2,38) = 45.75, *MSE* = 23,542, *p* < 0.0001, GG-*ε* = 0.627, and the comparisons of the location task with the other tasks (*p*s < 0.0001) were significant. For the error rates, significant differences across the tasks were obtained, too, *F*(2,38) = 9.72, *MSE* = 8.68, *p* = 0.0007, GG-*ε* = 0.880. Error rates were significantly lower in the location task (2.63%) than in the pattern task (5.42%) and the shape task (4.73%), with *p*s ≤ 0.001.

These findings indicate that despite the spatially incompatible S–R mapping used in Experiment 3, the location task is still easier than the other two tasks. This suggests that the relevant factor behind the location task’s dominance is not compatibility on the element level, but rather compatibility on the set level (dimensional overlap). This finding is reminiscent of findings in the S–R compatibility literature, showing that the benefit of spatially compatible S–R mappings is basically lost in the context of mixed S–R mappings (i.e., when compatible and incompatible mappings are mixed, or when a location-relevant task is mixed with a location-irrelevant task, e.g., Proctor and Vu, 2006, for a review). In the present context, the data of Experiment 3 suggest that the potential benefit of spatial S–R correspondence was not fully shown in Experiments 1 and 2 because the mixed mappings might have suppressed this particular benefit. Presumably, had we included single-task control conditions, we might have found that the performance benefit in these blocks relative to the task-switching blocks would have been largest for the location task with the compatible mapping. However, in the absence of such single-task control conditions, we can only speculate that automatic response activation may not be the driving factor in the performance benefit of the location task relative to the other two tasks. That is, dimensional overlap more generally seems to matter.

In fact, in Experiment 3, RTs in the location task were not longer than in Experiment 2, in which the S–R mapping was compatible. RTs were even slightly shorter in Experiment 3 than in Experiment 2. If anything, then the RTs in the other two tasks increased in Experiment 3 compared to Experiment 2. However, these differences did not reach significance, *F*(2,76) = 2.54, *MSE* = 23,068, *p* = 0.107, GG-*ε* = 0.671, for the interaction Experiment × Task.

#### Backward Inhibition

As before, sequence had a significant effect, *F*(1,19) = 17.93, *MSE* = 677, *p* = 0.0004. With 24 ms, which is an increase of 4.65%, the n-2 repetition costs were again slightly larger for the location than for the other two tasks. However, the interaction Task × Sequence was not significant (*F* < 1). Also, with the proportional scores the differences did not reach significance, *F*(2,38) = 2.20, *MSE* = 18, *p* = 0.1259, GG-*ε* = 0.981.

In the error rates, small n-2 repetition costs were numerically present in all three tasks (with differences between ABA and CBA sequences of 0.28, 0.20, and 0.46% for the location, pattern, and shape tasks, respectively), which, however, did not reach significance, *F*(1,19) = 1.21, *MSE* = 2.43, *p* = 0.2847.

## Common Analysis of Experiments 1–3

Across all three experiments, the n-2 repetition costs were numerically larger for the location task, thus, supporting our hypothesis that dominant tasks are inhibited more strongly. However, except for Experiment 2, these differences were not significant. Given that the n-2 repetition costs here are relatively small (23 ms on average, compared to, e.g., 80 ms in Schuch and Koch, 2003, who used a different set of tasks), differences in these costs probably require a larger sample to be detected, that is, the failure of finding significant differences might be an issue of statistical power. We, therefore, ran analyses with a sample that includes all three experiments.

For the combined ANOVA (with experiment as factor), we directly tested the n-2 repetition costs in the location task against the average costs in the other two tasks. Both the test with the proportional costs as well as the test with the raw difference scores were significant, *F*(1,57) = 14.61, *MSE* = 23, *p* = 0.0003 and *F*(1,57) = 5.80, *MSE* = 424, *p* = 0.0193, respectively (no significant interactions with experiment, *p*s > 0.22). Thus, increasing the power by pooling the data across the three experiments (with 20 participants each) revealed significant differences in the size of the n-2 repetition costs.

## General Discussion

Flexible switching between tasks is assumed to be accompanied by inhibiting strong competitor tasks or tasks that were relevant previously. This inhibitory process is thought to reduce conflict allowing one to efficiently perform the currently relevant task (Mayr and Keele, 2000; Koch et al., 2010). Given this, it is plausible to assume that stronger or more dominant tasks require more inhibition than weaker and less dominant ones. Evidence regarding this postulated relation between the dominance pattern of the tasks and the mechanisms applied to control intertask interference is scarce, because in research on task switching it is common practice to aggregate across tasks. Therefore, we here systematically manipulated task dominance and assessed n-2 repetition costs as an empirical marker of inhibition separately for each task.

Across three experiments, participants performed a location discrimination task with strong S–R bindings (i.e., dimensional overlap between stimuli and responses, see Kornblum et al., 1990) substantially faster and more accurately than color/pattern discrimination and shape discrimination tasks, for which S–R mappings were arbitrary. Because stimuli were multidimensional, conflict in processing the color/pattern and shape tasks arises because of an irrelevant S–R overlap with the response mappings of the location task (i.e., the irrelevant stimulus dimension spatial location [left vs. right presented objects] overlaps with the relevant response dimension [left vs. right manual response]). Consequently, the location task was not only processed faster and more accurately, but also can be assumed to be dominant in the context of the other tasks, because of the interference it exerts (similar to the Simon effect, Simon, 1990).

Along with the clear dominance pattern in overall performance, n-2 repetition costs differed across the tasks, showing larger costs for the dominant location task than for the two weaker tasks. This finding suggests that the amount of inhibition is adjusted to a task’s dominance and, thus, extends our knowledge of inhibitory processes and their role for cognitive flexibility. Dominant tasks (or stimulus dimensions) such as the one used in our study, normally show strong interference on weaker tasks (cf. Simon effect, Simon, 1990; Lu and Proctor, 1995; see also Stroop effect, MacLeod, 1991, for review). In a task-switching situation, in which the dominant task/stimulus dimension was relevant recently, the potential source of task-set carry-over and interference seems to be counteracted by a relatively large degree of inhibition (see also Gade and Koch, 2005). In contrast, weaker and, thus, less interfering tasks seem to receive a smaller amount of inhibition. Although the observed differences in the size of the n-2 repetition costs were small and did not reach significance in each experiment, the basic pattern was observed in three experiments and thus proved replicable. Our data, thus, not only fit with previous evidence for an adjustment of the amount of inhibition to task difficulty (Arbuthnott, 2008), but extends this by providing evidence for a direct link between the dominance of a task and the amount of inhibition it receives to counteract the conflict it exerts.

The fact that the asymmetry in n-2 repetition costs are observed for different stimulus materials and tasks (see, e.g., Philipp et al., 2007; Declerck et al., 2015, for language switching; Arbuthnott, 2008, for task switching), in the first instance, provides strong evidence for the robustness and generality of this effect. Beyond that, it may also help to broaden our knowledge about the nature of inhibitory processes in task switching.

Specifically, as between-task interference in the various tasks typically used in task-switching studies arise in different ways, inhibition presumably also targets different aspects of processing, that is, the locus and focus of inhibition may differ across tasks and paradigms. For instance, Arbuthnott (2008) used digit-categorization tasks for which semantic aspects of a digit, such as magnitude or parity, are to be retrieved from long-term memory. In the present study, the stimuli for the color/pattern and shape tasks are multidimensional stimuli that contain an S–R overlap with the irrelevant stimulus dimension (left vs. right spatial location) overlapping with the relevant response dimension (left vs. right manual response). Accordingly, inhibition may be targeted at the irrelevant perceptual stimulus dimension location and its corresponding response. Conflict in the Simon task is assumed to result from an automatic response activation through a direct route that assesses long-term S–R associations (see, e.g., Hommel and Prinz, 1997; Proctor and Vu, 2006, for reviews). In this regard, inhibiting the location task in the present study might contain the suppression of this direct route.

One finding from our study seems to be particularly suggestive with regard to the suppression of the direct route. Comparing the results of Experiments 2 and 3 revealed that an incompatible mapping of stimuli and responses in the location task neither changed the dominance pattern nor the asymmetry of the n-2 repetition costs. As already discussed above, this reminds one of the often observed elimination of the S–R compatibility effect under mixed-task conditions (Proctor and Vu, 2006, for review). The most widely accepted account for this is that the direct response-selection route is suppressed when S–R mappings are mixed (e.g., Proctor and Vu, 2002). In the present study, the absence of a strong S–R compatibility effect in the location task may indicate that in this specific task-switching context we have used here, the translation of the relevant stimulus code into a response code via the direct route is suppressed (even when the mapping was compatible). Although this needs to be addressed in further studies (e.g., by including pure blocks and a within-subject manipulation of S–R compatibility), it provides a first hint that exactly this link to long-term representations is the locus of the inhibitory process when it comes to control potential conflict that arises from the location task.

The observed differences in the n-2 repetition costs as a function of task dominance were comparatively small and might, therefore, be of little importance in the microstructure of task switching. However, the n-2 repetition costs proper already proved to be rather small in the present study. Hence, it is not surprising that any difference in the size of the costs also turn out to be small. On the other hand, task dominance constitutes only one instance of a broader category of factors that determine the amount of inhibition needed to counteract conflict. As previous studies pointed out, factors such as the time elapsed between the tasks (e.g., Gade and Koch, 2005), the representational overlap of the response sets (Gade and Koch, 2007) as well as increased intertrial conflict (Grange and Houghton, 2010) are responsible for the magnitude of inhibition. Together these findings indicate that backward inhibition is a particularly flexible mechanism that is sensitive to many aspects of the context.

## Conclusion

The present study provides evidence that in task-switching situations a dominant task receives more inhibition than weaker ones presumably to counteract the potential source of task-set carry-over to subsequent trials. This finding fits with previous demonstrations that the degree of inhibition can vary with particular context demands. Extending these findings, the effects in the present study suggest a direct link between a task’s activation and the inhibition it receives. Inhibition as a means of cognitive flexibility, thus, is not a “blunt” mechanism enabled whenever interference is likely to occur, but adjusted in strength according to specific requirements of the context and the dominance dynamic of the tasks.

## Author Contributions

KJ, IK, and VH designed the study. KJ (assisted by VH) analyzed the data. KJ, IK, and VH discussed the results. KJ wrote the first draft of the manuscript. IK and VH commented on the draft. All authors approved the final manuscript. All authors agreed to be accountable for all aspects of the work in ensuring that questions related to the accuracy or integrity of any part of the work are appropriately investigated and resolved.

## Conflict of Interest Statement

The authors declare that the research was conducted in the absence of any commercial or financial relationships that could be construed as a potential conflict of interest.

## Footnotes

1 Note that we used the word “Typ” (type) instead of the perhaps more catchy word “Form” (form) for indicating the shape task. This was done to make the differentiation from the color-task indicating cue “Farbe” (color) easier.
